# Proto-oncoprotein Zbtb7c and *SIRT1* repression: implications in high-fat diet-induced and age-dependent obesity

**DOI:** 10.1038/s12276-021-00628-5

**Published:** 2021-05-20

**Authors:** Won-Il Choi, Jae-Hyun Yoon, Seo-Hyun Choi, Bu-Nam Jeon, Hail Kim, Man-Wook Hur

**Affiliations:** 1 Brain Korea FOUR Project for Medical Science, Department of Biochemistry & Molecular Biology, Yonsei University School of Medicine, 50-1 Yonsei-Ro, SeoDaeMoon-Ku, Seoul, 03722 Korea; 2 Graduate School of Medical Science and Engineering, Korea Advanced Institute of Science and Technology, DaeJeon, 34141 Korea; 3 KAIST Institute for the BioCentury, Korea Advanced Institute of Science and Technology, Daejeon, 34141 Korea

**Keywords:** Metabolomics, Transcriptional regulatory elements

## Abstract

Zbtb7c is a proto-oncoprotein that controls the cell cycle and glucose, glutamate, and lipid metabolism. Zbtb7c expression is increased in the liver and white adipose tissues of aging or high-fat diet-fed mice. Knockout or knockdown of Zbtb7c gene expression inhibits the adipocyte differentiation of 3T3-L1 cells and decreases adipose tissue mass in aging mice. We found that Zbtb7c was a potent transcriptional repressor of *SIRT1* and that *SIRT1* was derepressed in various tissues of *Zbtb7c*-KO mice. Mechanistically, Zbtb7c interacted with p53 and bound to the proximal promoter p53RE1 and p53RE2 to repress the *SIRT1* gene, in which p53RE2 was particularly critical. Zbtb7c induced p53 to interact with the corepressor mSin3A-HADC1 complex at p53RE. By repressing the *SIRT1* gene, Zbtb7c increased the acetylation of Pgc-1α and Pparγ, which resulted in repression or activation of Pgc-1α or Pparγ target genes involved in lipid metabolism. Our study provides a molecular target that can overexpress SIRT1 protein in the liver, pancreas, and adipose tissues, which can be beneficial in the treatment of diabetes, obesity, longevity, etc.

## Metabolic disease: SIRT1 gene, a potential therapeutic target

Targeting a regulatory DNA sequence linked to the repression of a critical enzyme during metabolic diseases could prove valuable for future therapies. The SIRT1 enzyme is involved in metabolic processes and stress resistance, and its dysregulation is linked to obesity and diabetes development. SIRT1 expression also decreases with aging and stress, but the precise regulation mechanisms are unclear. In experiments on aging mice and mice fed a high-fat diet, Man-Wook Hur at Yonsei University in Seoul, South Korea, and co-workers demonstrated that SIRT1 expression is repressed by a protein called Zbtb7c, which is highly expressed in fat and liver tissues. Aging mice without the Zbtb7c-encoding gene had less fatty tissue than controls. Zbtb7c represses the *SIRT1* gene by interacting with protein p53. A sequence critical to this repression mechanism may provide a therapeutic target.

## Introduction

Sirtuins (SIRT1-7) are NAD^+^-dependent enzymes that catalyze post-translational modifications of proteins. Sirtuins catalyze a range of deacetylation reactions, and some family members, namely SIRT2, 4, and 6 require nicotine adenine dinucleotide (NAD+) as a cofactor^1^. Together, these SIRTs and NAD+ regulate critical cellular pathways and are associated with aging and longevity. SIRT1 is the best-characterized member of the mammalian sirtuin family. SIRT1 deacetylases play roles in mitochondrial biogenesis, glucose and lipid metabolism, DNA repair, stress resistance, apoptosis, and inflammation^2^. Dysregulation of SIRT1 expression or function has significant implications in the development of obesity, diabetes, cancer, cardiac disease, neurodegenerative disease, metabolic dysregulation, etc.

Caloric restriction, fasting, and food withdrawal increased SIRT1 expression in several rodents and human tissues, such as white adipose tissue, liver, skeletal muscle, brain, and kidney. The *SIRT1* promoter contains two Hypermethylated in Cancer1 (HIC-1) response elements (HIC-1REs), an E2F1-binding element, and two p53-binding elements (p53REs), insulin receptor substrate-1-like binding sequences (IRS-1) and a FOXO1 forkhead-like consensus binding site (FKHD-L). SIRT1 induction is partially mediated by the interplay between cAMP response element-binding protein (CREB) and carbohydrate response element-binding protein (ChREBP)^3^. CREB and ChREBP independently bind to the *SIRT1* promoter. CREB activated by cAMP-dependent protein kinase (PKA) activates *SIRT1* transcription. In contrast, ChREBP is rephosphorylated and translocated into the nucleus and represses *SIRT1* transcription^3^. FOXO1 binds to IRS-1 and FKHD-L sites to activate rat *SIRT1* transcrption^4^. Upon acute nutrient withdrawal, FOXO3a interacts with p53, and the complex binds to p53-binding sites to activate *SIRT1* transcription^5^. p53 can be a transcription activator or repressor of the *SIRT1* promoter, depending on the presence of FOXO3a. It appears that p53 plays a critical role in the transcriptional regulation of *SIRT1* in response to various signals.

Interestingly, SIRT1 expression is decreased by aging and stress. An age-dependent decrease in SIRT1 expression in the liver is mediated by transcriptional repression by the C/EBPβ-HDAC1 complex^6^. C/EBPα activates *SIRT1* transcription during adipogenesis by binding to the C/EBP binding element^7^. SIRT1 protects cells from p53- and FOXO3-mediated apoptosis in response to oxidative stress^8,9^. NF-κB, which is a crucial stress response mediator, was shown to activate the *SIRT1* promoter, and SIRT1, in turn, inhibits NF-κB function by deacetylating NF-κB^10–12^.

Interestingly, the POZ domain and Krüppel (POK) family of transcription factors (HIC1, HIC2, and Zbtb7c) regulate the expression of *SIRT1*. HIC1 (hypermethylated in cancer 1) complexed with CtBP represses *SIRT1* transcription^13^. When DNA is damaged, HIC-1 and p53 jointly repress the *SIRT1* gene by binding to two HIC-1REs and two p53REs, respectively^14^. Recently, HIC2 was shown to activate the transcription of *SIRT1*^15^. Zbtb7c, encoded by *Zbtb7c*, represses transcription by recruiting cofactor complexes (e.g., HDAC, NuRD, and BCoR complexes)^16–19^. Immunohistochemical analyses of Sirt1 expression in various tissues of *Zbtb7c*-KO mice showed that Sirt1 is derepressed in all tissues tested (unpublished data), suggesting that Zbtb7c may be a transcriptional repressor of *Sirt1*.

Zbtb7c is a proto-oncoprotein that increases cell proliferation by repressing the transcription of *p21Cdkn1a* by inhibiting p53 or Miz-1^17,18^. Zbtb7c increases *Fasn* transcription^20^. Recently, we showed that Zbtb7c is vital in gluconeogenesis during fasting and in glutamine metabolism in cancer cells^21,22^. These results suggested that Zbtb7c might be an essential regulator of metabolism.

Despite numerous publications on Sirtuin family proteins, our understanding of these proteins and the molecular mechanism by which dietary conditions and aging regulate the *SIRT1* gene is still limited, and more studies are necessary. In this study, we found that Zbtb7c plays a vital role in lipid metabolism by repressing *SIRT1* transcription through a unique mechanism that involves molecular interaction between Zbtb7c and p53 and switching the p53-interacting transcription coregulators from p300 to mSin3A-HDAC1/3 at p53RE2.

## Materials and methods

### Mice

Animal experiments were approved by the Committee on Animal Investigations of the Department of Laboratory Animal Resources, Yonsei Biomedical Research Institute, Yonsei University College of Medicine. C57BL/6J mice were purchased from Charles River Japan (Yokohama, Japan). The mice were housed in a specific pathogen-free barrier facility under a 12-h light–dark cycle. Food and water were provided *ad libitum*. At 12 weeks of age, mice were fed a high-fat diet (HFD, 60% fat calories) for 14 weeks. The mice were euthanized by cervical dislocation.

### Plasmids, antibodies, and reagents

The pcDNA3.0-FLAG-Zbtb7c and pcDNA3.1-Zbtb7c constructs were prepared by cloning full-length cDNA into pcDNA3.0 or pcDNA3.1 (Invitrogen, CA). The pGL2-*SIRT1*-Luc human *SIRT1* promoter-luciferase reporter gene fusion construct was generated by cloning *SIRT1* promoter DNA (−1250 ~ +79 bp) into pGL2-Basic (Promega, WI). The pcDNA3-p53, pcDNA3-Sp1, and corepressor expression vectors have been reported elsewhere^4,5^. All plasmid constructs were verified by DNA sequencing. Descriptions of the recombinant adenovirus shRNA and siRNA against Zbtb7c mRNA have been reported elsewhere^18,21^. Antibodies against p53, p300, SIRT1, AMPK, phosphorylated APMK, PGC-1, PPARγ, Zbtb7c, acetylated lysine, GAPDH, FLAG-Tag, Myc-Tag, Ac-H3, Ac-H4, and mSin3A were purchased from Upstate (Charlottesville, VA), Chemicon (Temecula, CA), Cell Signaling Technology (Beverly, MA), Calbiochem (San Diego, CA), and Santa Cruz Biotech (Santa Cruz, CA). A rabbit polyclonal antibody against Zbtb7c was prepared in-house using recombinant GST-POZ (a.a. 1–120) and purified using Affi-Gel 10 (Bio-Rad, CA). Most of the chemical reagents were purchased from Sigma (St. Louis, MO).

### Cell cultures

Various cell types were cultured in the media recommended by ATCC. Zbtb7c^+/+^ and Zbtb7c^−/−^ mouse embryonic fibroblasts (MEFs) were prepared through a standard protocol and cultured in Dulbecco’s Modified Eagle Medium (DMEM) (Gibco-BRL, MD) supplemented with 10% fetal bovine serum (FBS) (Gibco-BRL, MD).

### Serum lipid analysis

Cholesterol, triacylglycerol, and total lipids were measured by an enzymatic colorimetric or a colorimetric method using an autoanalyzer (BS-400; MINDRAY, China).

### Transcription analysis of *SIRT1* promoters

The promoter-reporter fusion construct pGL2-*SIRT1*-Luc and the expression vectors pcDNA3-FLAG-*Zbtb7c*, pcDNA3.1-*Zbtb7c*, pcDNA3-*p53*, pcDNA3.1-*HIC-1*, and pCMV-*LacZ*, in various combinations were transiently transfected into cells using Lipofectamine Plus reagent (Invitrogen, CA) and analyzed as described elsewhere^15^.

### Reverse transcription and quantitative PCR (RT-qPCR) of Zbtb7c, SIRT1, and SIRT1 target mRNA

Total RNA was isolated from the various cells using TRIzol reagent (Invitrogen, CA). cDNA was synthesized from 5 µg of total RNA, random hexamers (10 pmol), and Superscript reverse transcriptase II (200 units) (Invitrogen, CA). RT-qPCR was performed using SYBR Green Master Mix (Applied Biosystems, CA) in an Applied Biosystems®7500 Real-Time PCR system. The oligonucleotide primers used for the qPCR assays are listed in Table 1.

**Table 1:** List of oligonucleotides used in the RT-qPCR analysis of mRNA expression levels and the ChIP qPCR analysis.

	Forward	Reverse
*Gene*		
Zbtb7c	5′-CCCATCTGCCACAAAGTCATC-3′	5′-TGGTGCACATGTATGGCTTCTC-3′
Ap2	5′-AAGGTGAAGAGCATCATAACCCT-3′	5′-TCACGCCTTTCATAACACATTCC-3′
Cd36	5′-AAGCTATTGCGACATGATT-3′	5′-GATCCGAACACAGCGTAGAT-3′
Fasn	5′-GCTGGCATTCGTGATGGAGTCGT-3′	5′-AGGCCACCAGTGATGATGTAACTCT-3′
Scd1	5′-ACGCCGACCCTCACAATTC-3′	5′-CAGTTTTCCGCCCTTCTCTTT-3′
Fsp27	5′-GCCACGCGGTATTGCCAGGA-3′	5′-GGGTCTCCCGGCTGGGCTTA-3′
G6pc	5′-AAGCCAACGTATGGATTCCG-3′	5′-ACAGCAATGCCTTGACAAGACT-3′
Pepck	5′-TCTCTGATCCAGACCTTCCAA-3′	5′-GAAGTCCAGACCGTTATGCAG-3′
Cpt1a	5′-GATGAACTTCTTCTTCCAGGAGTGC-3′	5′-ATGGCAGAGGCTCACCAAGC-3′
Cyp4a10	5′-AGAGTCTGGTGCAAACCTGG-3′	5′-CCCTGATGGACGCTCTTTAC-3′
Mcad	5′-GATGCATCACCCTCGTGTAAC-3′	5′-AAGCCCTTTTCCCCTGAAG-3′
Pdk4	5′-AGGGAGGTCGAGCTGTTCTC-3′	5′-GGAGTGTTCACTAAGCGGTCA-3′
Pparg	5′-GCATGGTGCCTTCGCTGA-3′	5′-TGGCATCTCTGTGTCAACCATG-3′
Pgc-1	5′-GGCACGCAGCCCTATTCA-3′	5′-CGACACGGAGAGTTAAAGGAAGA-3′
Human SIRT1	5′-GTATTTATGCTCGCCTTGCTG-3′	5′-TGACAGAGAGATGGCTGGAAT-3′
Mouse SIRT1	5′-GATCCTTCAGTGTCATGGTT-3′	5′-GAAGACAATCTCTGGCTTCA-3′
18S	5′-ATTGGAGCTGGAATTACCCGC-3′	5′-CGGCTACCACATCCAAGGAA
*Promoter*		
Pdk4	5′-ATTGGCTACTGTAAAAGTCCCG-3′	5′-TCCCAGGTCGCTAGGACTTCAG-3′
Mcad	5′-CCTTGCCCGAGCCTAAAC-3′	5′-GTCTGGCTGCGCCCTCT-3′
aP2	5′-AATGTCAGGCATCTGGGAAC-3′	5′-GACAAAGGCAGAAATGCACA-3′
Cd36	5′-ATTTGTGGTTGGTTGCCAAG-3′	5′-AGGTGATGGGTCTTCACCAG-3′

### Western blot analysis

Cells were harvested and lysed in RIPA buffer (50 mM Tris-HCl, pH 8.0; 1% NP-40; 0.25% sodium deoxycholic acid; 150 mM NaCl; 1 mM EGTA; and complete mini-protease cocktail). Cell extracts (40 µg) were separated using 10% sodium dodecyl sulfate-polyacrylamide gel electrophoresis (SDS-PAGE), transferred onto Immun-Blot^TM^ polyvinylidene difluoride (PVDF) membranes (Bio-Rad, CA), and blocked with 5% skim milk (BD Biosciences, MD). The blotted membranes were incubated with antibodies against His-tag, Myc-tag (Cell Signaling, MA), FLAG-tag (Sigma, MO), GAPDH (Chemicon, CA), p53, HIC-1, Zbtb7c, mSin3A, and SIRT1 and then incubated with either an anti-mouse or anti-rabbit secondary antibody conjugated to HRP (Vector Laboratory, CA). Protein bands were visualized using an ECL solution (PerkinElmer, CA).

For western blot analysis of mouse tissues, frozen tissue samples were homogenized, and tissue lysates were extracted by incubating cells in RIPA buffer (25 mM Tris-HCl, pH 7.6; 150 mM NaCl; 1% NP-40; 1% sodium deoxycholate; and 0.1% SDS) plus protease inhibitors (Roche). Supernatants were collected following a brief centrifugation, and protein concentrations in the supernatants were measured using a BCA protein assay kit (Thermo Scientific, Rockford, IL, USA). The lysates were then mixed with equal volumes of 2× Laemmli buffer (4% SDS, 20% glycerol, 10% 2-mercaptoethanol, 0.01% bromophenol blue, and 120 mM Tris-HCl, pH 6.8) and boiled for 10 min at 95 °C. Next, the protein samples were separated by SDS–PAGE and transferred to a PVDF membrane (Millipore). After blocking in a 5% skim milk solution (Sigma), the membranes were incubated with the following specific primary antibodies: anti-ZBTB7c antibody (diluted 1:1000, Biorbyt orb349355), anti-p53 antibody (diluted 1:1000, BD Pharmingen^TM^ 554157), and anti-Actin antibody (diluted 1:1000, Cell Signaling #3700). The membranes were then washed with 1 × TBST and incubated with an anti-rabbit IgG horseradish peroxidase-linked antibody or anti-mouse IgG antibody. The detection of each protein was performed using SuperSignal West Pico Chemiluminescent Substrate (Thermo Scientific) according to the manufacturer’s instructions. Signals were captured by a ChemiDoc MP system (Bio-Rad). Blots of tissue and cell lysates were quantified using ImageJ (NIH).

### Coimmunoprecipitation/western blot assays

Protein extracts from liver tissue were washed, pelleted, and resuspended in lysis buffer supplemented with protease inhibitors (20 mm Tris-HCl, pH 7.5; 150 mm NaCl; 10% glycerol; 1% Triton X-100; complete mini-protease mixture (1 tablet/50 ml, Roche Applied Science)). Cell lysates were precleared, and the supernatants were incubated overnight with an anti-p53 antibody on a rotating platform at 4 °C and then incubated with protein A/G PLUS-Agarose beads (Santa Cruz, sc-2003). The beads were collected, washed, and resuspended in equal volumes of 5× SDS loading buffer. The immunoprecipitated proteins were separated using 8 and 10% SDS-PAGE. Western blot analysis was performed with the appropriate antibodies as described above.

### Chromatin immunoprecipitation and quantitative PCR (ChIP-qPCR) assays

ChIP assays were performed to assess in vivo Zbtb7c binding to distinct gene promoter regions. The primers used are listed in Supplementary Table 1. NIH3T3 and MEFs were transfected with increasing amounts of Zbtb7c expression vector, pcDNA3.0-FLAG-Zbtb7c, or pcDNA3.1-Zbtb7c. By ChIP assays, the molecular interactions between Zbtb7c and p53 and histone modifications at the endogenous *SIRT1* promoter were analyzed. The levels of FLAG-tagged Zbtb7c binding at the p53REs were analyzed using an anti-FLAG antibody (Sigma, Cell Signaling). The levels of Zbtb7c and p53 protein binding to the endogenous *SIRT1* promoter were analyzed by ChIP using polyclonal antibodies against Zbtb7c and p53 (Santa Cruz, CA). The following oligonucleotide primer sets were designed to amplify the regions flanking the p53-binding sites within the *SIRT1* promoter and acetylated histones H3 and H4 (−278 to −97 bp): forward 5′-CACTACACGCTCGCCACAAAGAGG-3′, and reverse 5′-GGAGATTTAAACCCCATCACGTGACC-3′.

### Immunoprecipitation assays

NIH3T3 cells, HCT116 p53^+/+^ cells, HCT116 p53^−/−^ cells, and MEFs were harvested and resuspended in lysis buffer (20 mM Tris-HCl, pH 7.5; 150 mM NaCl; 10% glycerol; and 1% Triton X-100) supplemented with protease inhibitors. Cell lysates were precleared, supernatants were incubated overnight with antibodies against Zbtb7c, p53, Sp1, Myc, or p300 or a corepressor on a rotating platform at 4 °C and then incubated with protein A-Sepharose Fast Flow beads. The beads were collected, washed, and resuspended in an SDS loading buffer. The immunoprecipitated proteins were separated with 10–12% SDS-PAGE, and western blot assays were performed as described above.

### Oligonucleotide pull-down assays

NIH3T3 and HEK293 cells were lysed in HKMG buffer (10 mM HEPES, pH 7.9; 100 mM KCl; 5 mM MgCl_2_; 10% glycerol; 1 mM DTT; and 0.5% NP40). Cell extracts were incubated with 1 μg of biotinylated double-stranded oligonucleotides (p53RE1 and p53RE2) for 16 hr. The oligonucleotide sequences were as follows (only the top strands are shown): p53RE1, 5′-AACAGCCTCCGCCCGCCA**CGTG**ACCCGTAGTGTT-3′, and p53RE2, 5′-TGTTGTGGTCTGGCCCG**CGTG**GGTGGCGGGAGCG-3′). The core p53-binding sequences are underlined. The core CGTG sequence was mutated in the pGL2-*SIRT1*-Luc reporter mutant plasmids (Fig. 3b). The mixtures of cell lysate and biotinylated oligonucleotide were incubated with streptavidin-agarose beads for 2 h, washed with HKMG buffer, and precipitated by centrifugation. The precipitates were analyzed by western blotting using antibodies against Zbtb7c, p53, and GAPDH, as described above.

### Statistical analysis

Student’s *t* test was used for statistical analyses.

## Results

### Zbtb7c regulates the transcription of *SIRT1*, *Pparγ*, and *Pgc-1α* and various genes encoding lipid metabolism enzymes

**Fig. 1:**
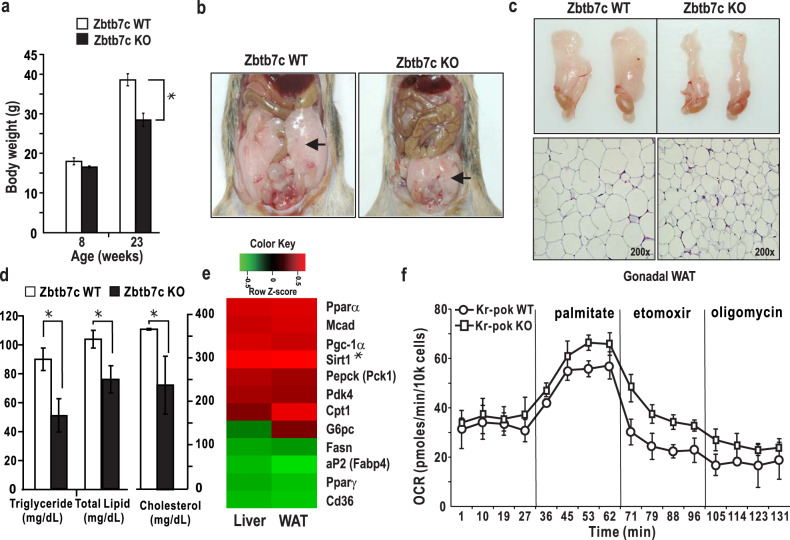
*Zbtb7c*-KO mice show reduced adipose tissue, and Zbtb7c inhibits fatty acid oxidation by regulating the expression of lipid metabolism genes. **a** Body weight of WT and *Zbtb7c*-KO mice at 8 and 23 weeks. In aging *Zbtb7c*-KO mice, there was a significant decrease in body weight. **b** Abdominal adipose tissue of 23-week-old WT and *Zbtb7c* KO mice. **c** Comparison of epididymal WAT from WT and *Zbtb7c*-KO mice. **d** Blood serum levels of triglycerides, total lipids, and cholesterol in WT and *Zbtb7c*-KO mice—23 weeks after birth (*n* = 6). **e** Heat map of differential mRNA expression of liver and adipose tissue in WT and *Zbtb7c* KO mice as determined by microarray analysis. **f** Extracellular flux (XF) analysis of mitochondrial palmitate metabolism. The oxygen consumption rate (OCR) of *Zbtb7c*-KO mouse MEFs in the presence of exogenous palmitate was increased, suggesting elevated β-oxidation of fatty acids by exogenous palmitate.

Previously, we found that Zbtb7c regulates the expression of fatty acid synthase, a key enzyme of fatty acid synthesis, suggesting that Zbtb7c plays a vital role in lipid metabolism^20^. Interestingly, in the present study, abdominal adipose tissue was significantly decreased in aging *Zbtb7c*-KO mice (Fig. 1a, b). Histological sections of gonadal WAT from the *Zbtb7c*-KO mice showed adipocytes that were smaller than those in the control mice (Fig. 1c). We also found significant decreases in triglycerides, total lipids, and serum cholesterol in the blood of the *Zbtb7c*-KO mice (Fig. 1d).

Microarray analyses of differentially expressed genes in the liver and WAT tissues of the *Zbtb7c-*KO mice showed changes in the expression of genes important in lipid metabolism (Fig. 1e and Supplementary Fig. 1). In *Zbtb7c*-KO mice, the mRNA expression of two critical metabolic regulators, *Pparγ* and *Pgc-1α*, and several other genes involved in fatty acid metabolism (*Ap2*, *Scd1*, *Cd36*, and *Fans*), adipocyte differentiation (*Pparγ*), and gluconeogenesis (*G6pc* and *Pepck*) were decreased. In contrast, the expression of genes involved in fatty acid β-oxidation or mitochondrial biogenesis (*Cpt1a*, *Cyp4a10*, *Mcad*, and *Pgc-1α*) was increased. In particular, in both liver and WAT tissues, the expression of *Pparγ* was downregulated, and that of *Pgc-1α* was significantly upregulated. Moreover, the expression of SIRT1, an essential regulator of glucose production in the liver (via TORC2, PGC-1α, and FOXO1), and lipid mobilization and metabolism in adipose tissue (through PPARγ and LXR) were significantly increased in *Zbtb7c-*KO mice (Supplementary Fig. 1).

Using western blot assays, we also analyzed the expression levels of signaling molecules upstream of Pgc-1α and Pparγ, such as Ampk, phosphorylated Ampk, Akt, and phosphorylated Akt in the liver and WAT. While the total expression of Ampk remained unchanged, that of phosphorylated Ampk, an activated form of Ampk that stimulates β-oxidation and ketogenesis and inhibits fat synthesis^23^, was significantly increased in the *Zbtb7c*-KO mice (Supplemental Fig. 1b, d). The expression of *Pgc-1α* was also upregulated, as expected. We also analyzed the expression of Akt and phosphorylated Akt in WAT tissues. Total Akt expression was similar in the WT and knockout mice. Nevertheless, the expression of phosphorylated Akt, a multifunctional kinase that plays critical roles in the inhibition of lipolysis and synthesis of fatty acids^23–25^, was significantly decreased by *Zbtb7c* knockout. These results suggest that Zbtb7c plays an essential role in lipid metabolism by regulating the expression and activity of upstream signaling molecules such as Ampk and Akt and the transcription of *SIRT1* and *Pparγ, Pgc-1α*, genes encoding lipid metabolism regulators in the liver and WAT.

Because the expression of the genes involved in fatty acid β-oxidation was increased in *Zbtb7c*-KO mice, we investigated the oxygen consumption rates (OCRs) of *Zbtb7c* WT and KO MEFs using a Seahorse XF24 Flux Analyzer. *Zbtb7c*-KO MEFs treated with palmitate had a higher baseline OCR. The *Zbtb7c*-KO MEFs showed an increase in the palmitate-induced OCR, indicating higher activity of fatty acid oxidation than induced in the WT *Zbtb7c* MEFs (Fig. 1f).

### Zbtb7c downregulates the transcription of *SIRT1* and stimulates adipocyte differentiation

**Fig. 2:**
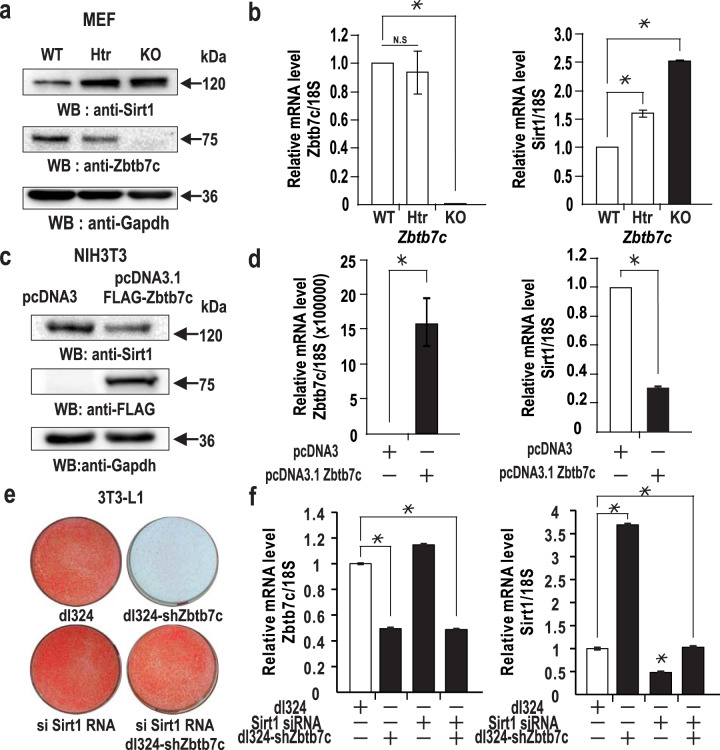
Zbtb7c represses *SIRT1* transcription and induces adipocyte differentiation. **a**, **b** qRT-PCR and western blot analyses of SIRT1 and Zbtb7c expression in WT and *Zbtb7c*-KO MEFs. **c**, **d** qRT-PCR and western blot analyses of SIRT1 and Zbtb7c expression in NIH3T3 cells transfected with the Zbtb7c expression vector. **e** Zbtb7c promoted adipocyte differentiation and/or lipid synthesis via *SIRT1* repression. 3T3-L1 preadipocytes were infected or transfected with recombinant *Zbtb7c* shRNA adenovirus and/or SIRT1 siRNA, cultured in differentiation medium for 14 days, and stained with oil red O reagent. **f** qRT-PCR analysis of SIRT1 and Zbtb7c expression in 3T3-L1 cells treated as in (**e**).

The expression of *Pgc-1α* was upregulated and that of *Pparγ* was downregulated in the *Zbtb7c*-KO mice. SIRT1 expression was derepressed in the liver and WAT of the *Zbtb7c*-KO mice. These changes were likely to increase fatty acid oxidation and reduce fat mass. Considering that the *Pgc-1α* and *Pparγ* genes are downstream of SIRT1 and that a negative correlation was found between SIRT1 and Zbtb7c expression, we investigated whether Zbtb7c can repress human *SIRT1* transcription and thereby regulate Pgc-1α and Pparγ expression or activity in three different mouse cell types: MEFs, NIH3T3 cells, and differentiating NIH3T3-L1 cells. In MEFs with the *Zbtb7c* gene knocked out, SIRT1 expression was higher than that in WT MEFs (Fig. 2a, b). While ectopic Zbtb7c repressed endogenous *Sirt1* expression in NIH3T3 cells, knockdown of Zbtb7c expression by infection with recombinant adenovirus expressing shZbtb7c RNA increased endogenous Sirt1 expression (Fig. 2c, d). We observed similar results in NIH3T3-L1 cells treated with adipocyte differentiation inducers.

Because SIRT1 inhibits adipocyte differentiation, we tested whether Zbtb7c can promote adipocyte differentiation by repressing *SIRT1* expression. Knockdown of Zbtb7c expression derepressed or increased *Sirt1* mRNA expression by 3.7-fold and inhibited adipocyte differentiation (Fig. 2e, f right). Knockdown of *SIRT1* expression enhanced adipocyte differentiation (Fig. 2e). Knockdown of both *Zbtb7c* and *SIRT1* resulted in SIRT1 mRNA expression and adipocyte differentiation similar to those of the control cells, suggesting that Zbtb7c represses SIRT1 expression and thereby affects adipocyte differentiation (Fig. 2e, f).

### Zbtb7c represses *SIRT1* transcription

**Fig. 3:**
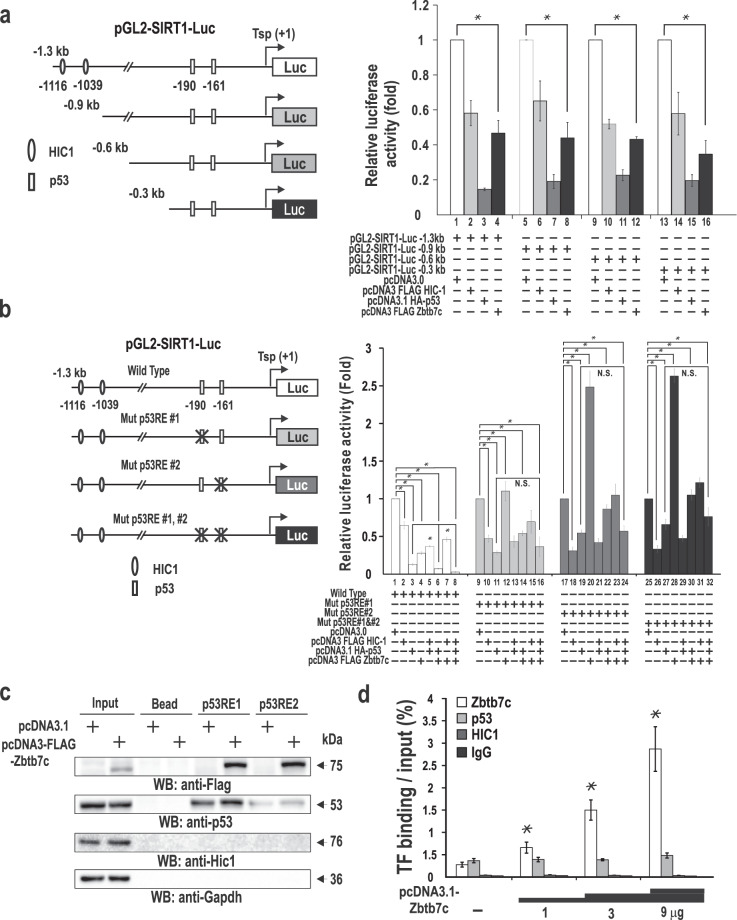
Zbtb7c represses *SIRT1* transcription by binding to p53REs. **a** Zbtb7c represses *SIRT1* transcription. Transient transcription assays of the four pGL2-*SIRT1*-Luc reporter construct in HEK293A cells. The Zbtb7c expression vector and reporter construct were transiently cotransfected, and luciferase activities were measured. Averages of three independents assays are shown. Error bars, standard deviations. **b** Site-directed mutagenesis of p53RE1 and/or p53RE2 in pGL2-*SIRT1*-Luc-1.3 kb. Transient transfection and transcription assays were performed to identify the critical elements in transcriptional repression of the *SIRT1* promoter by Zbtb7c. p53RE2 was critical to transcriptional repression by Zbtb7c. **c** Oligonucleotide pull-down assays of Zbtb7c binding to the p53REs of the *SIRT1* promoter in NIH3T3 cells. The pulled-down proteins were then analyzed by western blotting using the antibodies indicated. Zbtb7c bound to p53REs (equally well to both elements) and increased p53 binding to these elements. **d** qChIP assays of Zbtb7c, endogenous Hic-1, p53, and ectopic Zbtb7c binding to the p53REs of the *SIRT1* promoter in NIH3T3 cells. On p53RE regions, Zbtb7c binding was increased in a dose-dependent manner, but p53 binding remained constant. The antibodies used were anti-FLAG (for FLAG-tagged Zbtb7c), anti-p53, anti-Hic-1, or control IgG antibodies. Error bars, standard deviations.

Because SIRT1 and Zbtb7c expression showed a negative correlation, we investigated whether Zbtb7c can repress the transcription of the human *SIRT1* promoter. By performing transient transfection and subsequent transcription assays of four promoter and reporter gene fusion constructs (pGL2-*SIRT1*-Luc) (−1.4, −0.9, −0.6, and −0.3 kb sequences 5′-upstream from translation start site) in HEK293 cells, we investigated whether HIC1, p53, and Zbtb7c regulate *SIRT1* transcription. HIC1 repressed expression of the *SIRT1* promoter by 50–60% (Fig. 3a lanes 2, 6, 10, and 14). Zbtb7c also repressed reporter expression by 55–65% (Fig. 3a lanes 4, 8, 12, and 16). p53 potently repressed the transcription of pGL2-*SIRT1*-Luc, by more than 85% (Fig. 3a lanes 3 7, 11, and 15). We noticed that transcriptional repression by Zbtb7c was slightly more intense as the 5′-upstream sequence length was shortened, and transcriptional repression by p53 was somewhat weakened with the shortening of the sequences. Nonetheless, the data suggested that the proximal promoter region (bp, −300 ~ +79) was sufficient for transcriptional repression by p53 and/or Zbtb7c (Fig. 3a). Interestingly, the short *SIRT1* proximal promoter region contains two p53REs (p53RE1 and p53RE2, which are −188 ~ −181 bp and −159 to ~−152 bp from the ATG translation start codon, respectively). We meticulously investigated how the *SIRT1* promoter was regulated by HIC-1, p53, or Zbtb7c or by their combination. Zbtb7c enhanced transcriptional repression of pGL2-*SIRT1*-Luc WT through p53 (Fig. 3b lanes 3, 6), and this construct was further repressed by HIC1 (Fig. 3b lanes 3, 6, 8).

To investigate the function of p53REs in transcriptional regulation by the aforementioned factors (Zbtb7c, p53, and HIC1), we introduced mutations at p53REs by site-directed mutagenesis. The core CGTG sequences of either p53RE1 (−188 ~ −181 bp from the ATG translation start codon; 5′-ACGTGACC-3′) and/or p53RE2 (−159 ~ −152 bp in the core sequence 5′-GCGTGGGT-3′) were replaced with AGAG. Mutations in p53RE1 and/or p53RE2 profoundly affected the transcriptional regulation of SIRT1 by p53, Zbtb7c, and HIC1 (Fig. 3b). The mutation of p53RE1 abolished transcriptional repression that had been induced by Zbtb7c, and interestingly, the mutation of p53RE2 resulted in potent transcriptional activation (2.5-fold) of the SIRT1 promoter by Zbtb7c. The results suggested that both p53RE1 and p53RE2 were involved in transcriptional repression by p53 and Zbtb7c and suggested that p53RE2 was more critical in transcriptional repression by Zbtb7c. In the mutant constructs, transcriptional repression enhancement of the promoter by coexpressed p53 and Zbtb7c was also lost and attenuated transcription repression that had been induced by p53 approximately one fold (Fig. 3b, lanes 11 and 14 for the p53RE mutant 1 and lanes 19 and 22 for the p53RE mutant).

Mutation of p53RE1 and/or p53RE2 in the *SIRT1* promoter resulted in weaker transcriptional repression by p53 than observed for the WT promoter. The mutation of p53RE2 resulted in greater derepression. These data suggested the importance and nature of p53 binding at p53RE1 and p53RE2. p53 binding to both p53RE1 and p53RE2 was necessary and equally important in the transcriptional repression by p53. These data also suggested that p53RE1 was critical in the transcriptional repression by Zbtb7c, and mutation of the site abolished repression by Zbtb7c. p53RE2 was also crucial in transcriptional repression by p53 and in intense transcriptional repression by molecular interaction with Zbtb7c. Lack of p53 binding due to mutation created a situation in which the *SIRT1* promoter was potently activated by Zbtb7c. In the mutant reporter constructs, coexpression of p53 and Zbtb7c did not lead to enhanced transcriptional repression as observed for the WT promoter (Fig. 3b).

Intrigued by these observations, we investigated the molecular events (or DNA binding) among p53, Zbtb7c, and p53REs to understand how *SIRT1* gene transcription is regulated. Oligonucleotide pull-down assays also showed that ectopic Zbtb7c bound to both p53RE1 and p53RE2 with similar binding affinity. p53 bound more strongly to p53RE1 than to p53RE2, and ectopic Zbtb7c showed slightly increased p53 binding (Fig. 3c). These two different sets of data match closely, especially considering the nature of ChIP assays. The two p53REs are located very close to each other, and accordingly, primer sets amplified the DNA regions encompassing the p53RE1 and p53RE2 regions. p53RE1 appears to be the primary site for p53 binding, and the p53–Zbtb7c interaction seems to be important in Zbtb7c binding in the region (Fig. 3c)

ChIP assays of NIH3T3 cells transfected with increasing amounts of Zbtb7c expression vector showed a dose-dependent increase in Zbtb7c binding to the elements spanning the two p53REs, but p53 binding remained virtually unchanged, although p53 binding after 9 µg of Zbtb7c expression vector was transfected showed a weak increase. HIC1 showed little binding activity (Fig. 3d). The two different DNA-binding assays gave virtually identical results.

**Fig. 4:**
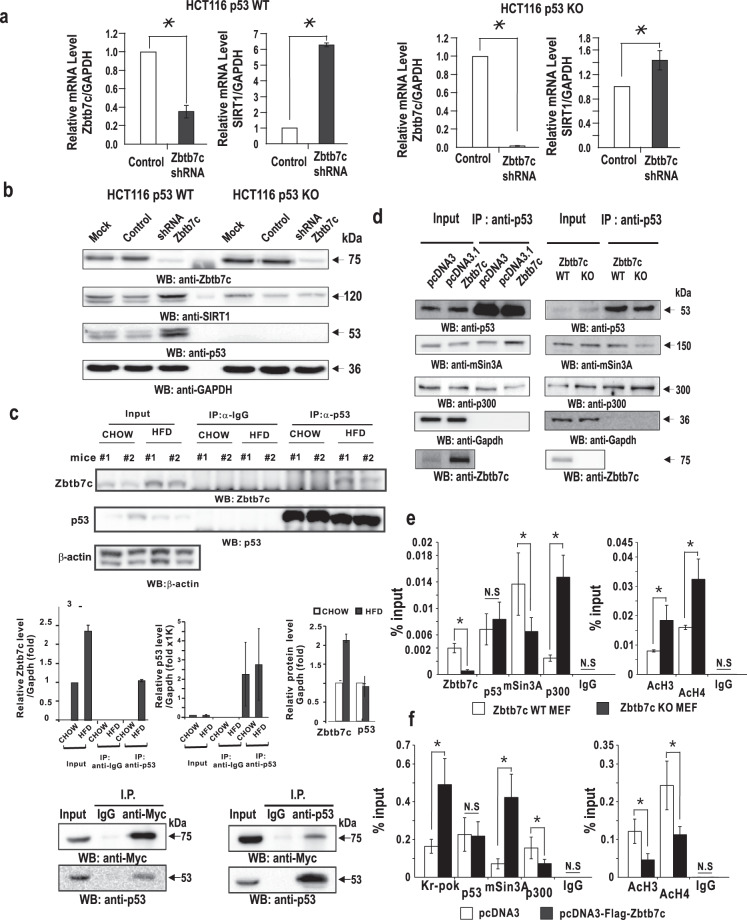
Transcriptional repression of *SIRT1* by Zbtb7c requires p53, and Zbtb7c increases binding of the mSin3A-HDAC1 complex to p53REs. **a**, **b** Zbtb7c represses *SIRT1* more strongly in WT p53 HCT116 cells than in p53-KO HCT116 cells. qRT-PCR and western blot analyses of Zbtb7c and SIRT1 expression in WT *TP53* and *TP*53-KO HCT116 cells. Cells infected with recombinant control adenovirus dl324 or recombinant dl324-Zbtb7c-shRNA. GAPDH, control. **c** *Top*. p53 and Zbtb7c interacted with each other. Coimmunoprecipitation of p53 and Zbtb7c and western blot assays. Liver extracts prepared from mice fed chow or HFD and NIH3T3 cell lysates with ectopic myc-Zbtb7c expression were subjected to coimmunoprecipitation with anti-Zbtb7c or anti-p53 antibody and to western blotting for detection of the interaction with p53 or Zbtb7c, respectively. *Middle*, histograms of the average protein band intensities of Zbtb7c, p53, and Gapdh. Western blot images were analyzed by ImageJ. *Bottom*, co-IP and western blot analyses of molecular interactions between ectopically expressed Myc-tagged Zbtb7c and endogenous p53. **d** Coimmunoprecipitation of p53, mSin3A, and p300 and western blot assays. Cell lysates were prepared from NIH3T3 cells transfected with ectopically expressed Zbtb7c. In addition, total cellular extracts of WT and *Zbtb7c*-KO MEFs were prepared. The cell lysates were coimmunoprecipitated with anti-p53 antibody and subjected to western blot assay for determination of the interaction with mSin3A and p300. The precipitates were analyzed by western blotting with the antibodies indicated. GAPDH, control. **e** ChIP-qPCR assays were performed to assess endogenous Zbtb7c, p53, mSin3a, and p300 binding to p53REs at the *SIRT1* promoter in wild-type and *Zbtb7c*-KO MEFs. **f** ChIP-qPCR assays were performed to assess endogenous Zbtb7c, p53, mSin3a, and p300 binding to p53REs at the *SIRT1* promoter in NIH3T3 cells transfected with FLAG-Zbtb7c. Error bars, standard deviations. In addition, the acetylation status of histone H3 and H4 near the proximal *SIRT1* promoter is shown. IgG, control ChIP antibody. Error bars, standard deviations.

Because p53 is a critical regulator of SIRT1 expression in response to various stresses, Zbtb7c may repress *SIRT1* transcription by modulating molecular interactions involving p53 at p53REs. We first tested whether endogenous *SIRT1* transcription repression by Zbtb7c is affected by the presence or absence of p53. The knockdown of Zbtb7c by shRNA resulted in strong derepression (an approximately 6-fold increase) of SIRT1 mRNA in WT p53-expressing HCT116 cells. However, knockdown of Zbtb7c in p53-null HCT116 cells resulted in only weak derepression (1.4-fold) of SIRT1 mRNA, suggesting that transcriptional repression by Zbtb7c strongly requires p53 activity (Fig. 4a). Western blot analyses of HCT116 cells showed similar results (Fig. 4b). These results indicated that Zbtb7c could potently repress *SIRT1* gene transcription in cells expressing p53 and weakly repress SIRT1 gene transcription in p53-null cells. p53 appeared to play a pivotal role in the repression of *SIRT1* by Zbtb7c.

### Zbtb7c interacts with p53, which increases p53 interaction with corepressor mSin3A–HDAC complex, not coactivator p300, decreasing the acetylation rate of histone H3 and H4 at the p53REs in the *SIRT1* proximal promoter

Interestingly, Zbtb7c more potently repressed the *SIRT1* gene promoter in cells expressing p53 than in cells not expressing p53. p53 is a transcriptional repressor of the *SIRT1* gene promoter. We suspected that Zbtb7c might have interacted with p53 and that this interaction led to more potent transcriptional repression of *SIRT1*. Co-IP and western blotting assays of the liver tissue extracted from mice fed an HFD and the cellular extracts prepared from cells transfected with the Myc-Zbtb7c and p53 expression vectors showed that the endogenous and/or ectopically expressed Zbtb7c and p53 proteins interacted with each other (Fig. 4c, top and bottom, and d).

We further investigated how Zbtb7c enhanced the transcriptional repression of the *SIRT1* promoter by p53. p53 was previously shown to repress *p21CDKN1A* transcription by interacting with the mSin3A-HDAC complex^18^. Interestingly, our co-IP assays showed that ectopic Zbtb7c increased the interaction between p53 and the mSin3A-HDAC complex but decreased the p53 interaction with p300 (Fig. 4d, left). the Co-IP assays of *Zbtb7c*-KO MEF extract performed with anti-p53 antibody also showed that the interaction of p53 with p300 was increased, and the interaction with the mSin3A-HDAC corepressor complex was weakened in the absence of Zbtb7c expression (Fig. 4d right).

ChIP assays of the binding of various factors (Zbtb7c, p53, mSin3A, and p300) in *Zbtb7c*-KO and WT MEFs indicated that, in the absence of Zbtb7c, the binding of mSin3A was decreased, and the binding of p300 was significantly increased. The binding of p53 was not affected (Fig. 4e). In addition, the ChIP assays of NIH3T3 cells transfected with the Zbtb7c expression vector showed that while ectopic Zbtb7c expression did not affect p53 binding, it increased mSin3A binding to p53RE sites and decreased p300 binding (Fig. 4f). These molecular changes induced by Zbtb7c decreased the acetylation of histones H3 and H4 around the p53REs of the *SIRT1* promoter, which decreased *SIRT1* transcription (Fig. 4e, f, right). Overall, these data suggested that Zbtb7c interacted with p53, bound to p53RE sites, and increased recruitment of the mSin3A-HDAC corepressor complex to potently repress *SIRT1* transcription.

### Zbtb7c increases acetylation of the transcription factors Pparγ and Pgc-1α by repressing SIRT1 expression and decreasing the binding of Pparγ or Pgc-1α to target genes

**Fig. 5:**
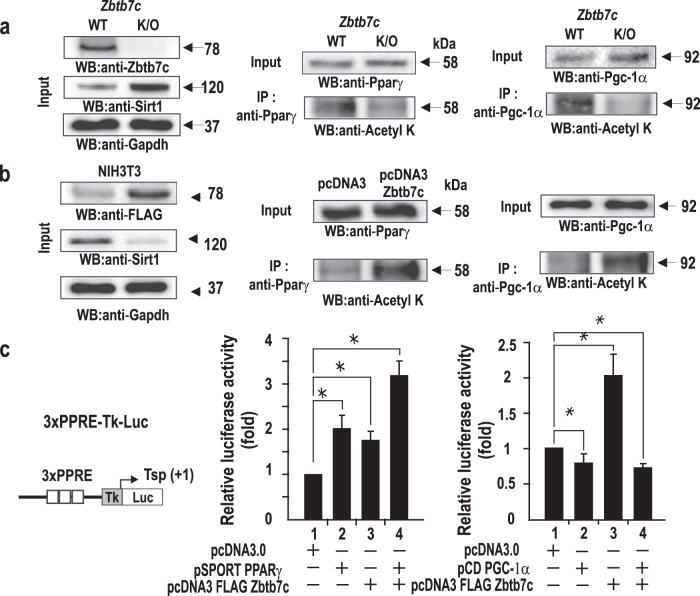
Zbtb7c leads to the acetylation of Pparγ and Pgc-1α by repressing *SIRT1*. **a** Immunoprecipitation and western blot analyses of acetylated Pparγ or Pgc-1α in WT and *Zbtb7c*-KO MEFs. **b** Immunoprecipitation and western blot analysis of acetylated Pparγ or Pgc-1α in NIH3T3 cells. The lysates of NIH3T3 cells with ectopic FLAG-Zbtb7c expression were immunoprecipitated with anti-Pparγ or Pgc-1α antibody and analyzed by western blotting with anti-FLAG, anti-Pgc-1α, anti-Pparγ, and anti-Gapdh antibodies. **c** Assays of transcriptional activity of Zbtb7c and Pparγ on pGL3-3x(PPRE)-TK-Luc in HEK293A cells. The Zbtb7c expression plasmid, *Pparγ* expression plasmid, and pGL3-3x(PPRE)-TK-Luc were transiently cotransfected, the cells were harvested after 48 h, and the luciferase reporter activity was measured. Error bars represent standard deviations. In addition, the transcriptional activity of Zbtb7c and Pgc-1α on pGL3-3x(PPRE)-TK-Luc in HEK293A cells is shown. Luciferase reporter activity was measured as described above.

Most reports support the idea that SIRT1 stimulates lipid metabolism. For example, SIRT1 was previously shown to regulate adipocyte differentiation and fatty acid oxidation by deacetylating lysine residues in Pparγ and Pgc-1α^26,27^. Because Zbtb7c repressed *SIRT1* expression, we examined whether Zbtb7c can increase the posttranslational acetylation of Pparγ or Pgc-1α. In NIH3T3 cells overexpressing Zbtb7c, lysine acetylation of Pparγ and Pgc-1α was increased, and lysine acetylation of Pparγ and Pgc-1α was decreased in the *Zbtb7c*-KO MEFs (Fig. 5a, b). These results indicated that Zbtb7c could have increased the acetylation of Pparγ or Pgc-1α through the repression of *SIRT1*.

**Fig. 6:**
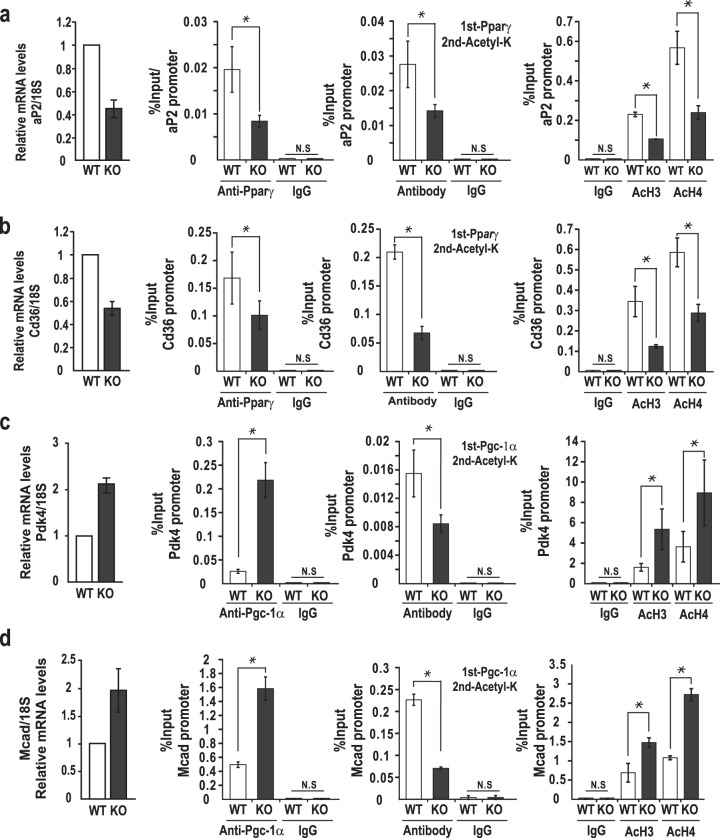
Zbtb7c target gene expression and ChIP assays of Zbtb7c binding at target gene promoters. **a**, **b** ChIP assays of Pparγ binding to the *aP2* and *Cd36* gene promoters. Lysates of WT and *Zbtb7c*-KO MEFs were immunoprecipitated with anti-Pparγ, anti-acetyl lysine, anti-AcH3, and Ac-H4 antibodies. IgG, control antibody. Error bars, standard deviations. **c**, **d** ChIP assay of Pgc-1α binding to the *Pdk4* and *Mcad* gene promoters. Lysates of WT and *Zbtb7c*-KO MEFs were immunoprecipitated with anti-Pgc-1α, anti-acetylated lysine, anti-AcH3, and Ac-H4 antibodies. IgG, control antibody. Error bars, standard deviations.

Furthermore, we tested whether Zbtb7c affected the transcription of the pGL3-3x(PPRE)-TK-Luc reporter plasmid carrying three Pparγ response elements in NIH3T3 cells. Transient transfection and reporter expression assays of the reporter plasmid (3xPPRE-Tk-Luc) showed that Pparγ or Zbtb7c activated transcription and that coexpression of Pparγ and Zbtb7c further increased transcription (Fig. 5c). The ChIP assays of Pparγ binding to the endogenous promoters of Pparγ target genes, including *aP2* and *Cd36*, showed an apparent decrease in *Zbtb7c* KO MEFs by Pparγ deacetylation by derepressed SIRT1. The ChIP assays also showed decreased acetylation of histones H3 and H4 at the Pparγ target gene promoters in *Zbtb7c*-KO MEFs, probably due to decreased Pparγ binding. This outcome may explain the low level of *aP2* and *Cd36* mRNA expression in *Zbtb7c*-KO MEFs and mouse liver and WAT tissues (Fig. 6a, b and Supplementary Fig. 1).

We also tested whether Zbtb7c affected the transcription of endogenous Pgc-1α target genes. Pgc-1α weakly repressed transcription of the reporter construct pGL3-3x(PPRE)-TK-Luc. Zbtb7c activated the reporter construct, probably by increasing lysine acetylation of Pgc-1α, via *SIRT1* gene repression (Fig. 5c right). The ChIP assays showed increased Pgc-1α binding at the endogenous promoters of its target genes, such as *Pdk4* and *Mcad*, in *Zbtb7c*-KO MEFs. The ChIP/re-ChIP assays performed using Pgc1α and anti-acetylated lysine antibodies showed decreased binding of acetylated Pgc-1α in the *Zbtb7c*-KO MEFs. The ChIP assays also showed increased acetylation of histones H3 and H4 in the endogenous Pgc-1α target promoters *Pdk4* and *Mcad* because of increased Pgc-1α binding (Fig. 6c, d). These results showed that Zbtb7c repressed the *SIRT1* gene. In the absence of Zbtb7c, lysine residues of Pgc-1α were deacetylated by derepression of SIRT1, which may explain the inhibition of adipocyte differentiation and fatty acid synthesis and/or increased fatty acid oxidation. These data may explain the apparent decrease in abdominal adipose tissue in *Zbtb7c*-KO mice (Fig. 1b).

### Zbtb7c expression is increased and SIRT1 expression is decreased in mice fed an HFD and aging mice

**Fig. 7:**
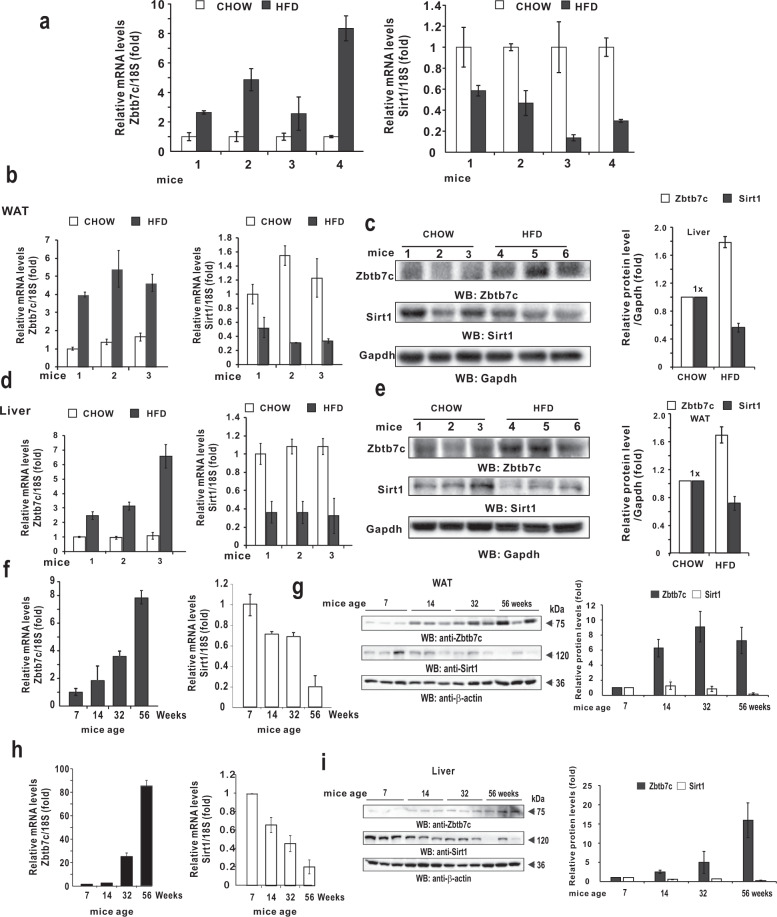
A high-fat diet and aging increase Zbtb7c expression but decrease SIRT1. **a** RT-qPCR analysis of Zbtb7c mRNA expression in the livers isolated from mice (*n* = 4) fed with chow or HFD. The mice fed with an HFD showed a lower level of Sirt1. **b–e** RT-qPCR analysis and western blot analyses of Zbtb7c and Sirt1 mRNA and protein expression in the liver and white adipose tissues isolated from the mice fed chow or an HFD. An HFD increased Zbtb7c expression but decreased Sirt1 expression. 18S RNA, RT-qPCR control; Gapdh, western control. On the right of (**c**) and (**e**), the histographs of the western blot protein (Zbtb7c, Sirt1 normalized with Gapdh) band intensities as analyzed by ImageJ are shown. **f**–**i** RT-qPCR analysis of Zbtb7c and Sirt1 mRNA expression in the eWAT and the liver isolated from mice (age 7, 14, 32, and 56 weeks). As the mice aged, Zbtb7c mRNA expression was increased, while Sirt1 mRNA expression was decreased. On the right, the western blots and histogram of Zbtb7c and Sirt1 protein expression are shown. 18S RNA, RT-qPCR control; β-actin, western control.

Having revealed the molecular mechanism of *SIRT1* transcription repression by Zbtb7c and p53, we investigated the physiological significance of our findings. First, we investigated the expression levels of Zbtb7c and SIRT1 in mice fed a normal chow diet and a HFD. Compared to the normal chow-fed mouse controls, the Zbtb7c level was increased by 2–8-fold and the SIRT1 mRNA level was downregulated by 59–85% in the HFD-fed mice (Fig. 7a, b. d). Additionally, at the protein level, Zbtb7c expression was increased and SIRT1 expression was downregulated in the eWAT and liver of the HFD-fed mice (Fig. 7c, e).

In addition, we investigated the expression patterns of *Zbtb7c* and Sirt1 during aging. As mice age into adulthood and reach the late adult period, *Zbtb7c* mRNA expression was increased up to 8–80-fold, and *Sirt1* was repressed by more than 80% in both WAT and liver tissues. We observed similar changes in protein expression (Fig. 7f–i). These data suggest that an HFD and aging can cause increased Zbtb7c expression and thereby decreased Sirt1 expression, which can affect diverse cellular processes (e.g., lipid metabolism) controlled by Sirt1 target genes, such as Pgc-1α, Foxo1, Foxo3, p53, Notch, Nf- κb, Hif-1α, and Srebp-1c.

## Discussion

SIRT1 has been studied for its role in caloric restriction, prevention of aging-related disease, and maintenance of metabolic homeostasis. Despite numerous publications on SIRT1, our understanding of the molecular mechanism of how the SIRT1 gene is regulated by diet conditions and aging remains limited. Activation of SIRT1 by the activator resveratrol or overexpression of SIRT1 can be beneficial for the treatment of metabolic and neurodegenerative diseases such as type 2 diabetes, obesity, Alzheimer’s disease, and Parkinson’s disease.

Previously, we and others showed that ZBTB protein family members such as HIC1 and HIC2 regulated transcription of the *SIRT1* gene. HIC1 is a transcriptional repressor that interacted with p53 and suppressed age-dependent cancer development in mice. The loss of HIC1 function promoted tumorigenesis by derepressing *SIRT1* and thereby attenuated p53 function by deacetylation, which increases cancer risk in mammals^14^. Recently, we found that HIC2, a homolog to HIC1 with differing intermediate domains, is a transcriptional activator of SIRT1 and may protect the heart from ischemia/reperfusion (I/R) injury^15^.

**Fig. 8:**
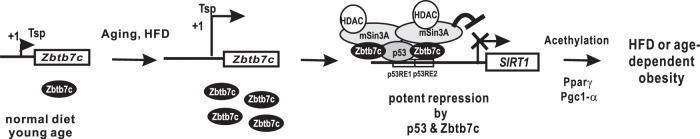
Hypothetical model of transcriptional regulation of Sirt1 by Zbtb7c and p53 in HFD-fed and aging mice. Aging and an HFD increased *Zbtb7c* transcription and protein expression. p53 played a critical role in transcriptional repression, as evidenced by knockdown of endogenous *Zbtb7c* in *TP53*-KO NIH3T3 cells showing weak derepression, whereas knockout of Zbtb7c in WT p53 cells showed strong derepression. p53 bound to p53REs (most likely at p53RE1) was critical to the transcriptional repression of Sirt1. p53 bound at p53RE1 interacted with Zbtb7c, and the complex recruited the corepressor-HDAC complex (e.g., mSin3A) to repress *SIRT1*. In the absence of Zbtb7c, p53 interacted with p300 and could activate Sirt1. Induction of Zbtb7c expression by aging or an HFD caused p53–Zbtb7c-corepressor/HDAC complex formation at p53RE1, and Zbtb7c triggered a coregulator switch. On the other hand, the involvement of p53 at p53RE2 was less clear because of weak binding to p53RE2. Zbtb7c was previously shown to lack the ability to bind p53RE, but interacted with p53 and formed a p53–Zbtb7c-corepressor/HDAC complex to repress *p21/CDKN1A*^18^. Although direct binding of Zbtb7c to p53RE2 is unlikely, Zbtb7c may have interacted with p53 and bound to p53RE2, resulting in the Zbtb7c-p53-corepressor/HDAC complex repressing *Sirt1*. The molecular interactions initiated by Zbtb7c overexpression led to *Sirt1* repression and subsequent acetylation of Pparγ and Pgc1-α, which may explain age-dependent or HFD-induced obesity.

Interestingly, the tumor suppressor protein p53 was previously shown to be a major regulator that activated or repressed the *SIRT1* gene depending on the physiological conditions. SIRT1, upon DNA damage stress, repressed transcription by binding to the two proximal p53-binding elements^14^. In contrast, upon acute nutrient withdrawal, p53 complexed with Foxo3a bound to the proximal p53-binding sites and activated mouse Sirt1, in which both p53RE1 and p53RE2 were equally important^5^. High nucleotide sequence homology of the proximal promoter regions (up to −220 bp regulatory region 5′-upstream from the ATG translation initiation codon) of human and mouse sirtuin genes suggested the functional importance of p53 elements and molecular events involving p53 and its interacting protein partners, such as Zbtb7c, in *SIRT1* gene transcription. Further, 5′-upstream regions beyond the −249 bp of the human *SIRT1* and mouse *Sirt1* genes (from the ATG translation initiation codon) showed little nucleotide sequence homology. Accordingly, the molecular events revealed at the human SIRT1 proximal promoter region (−248 bp from the ATG translation codon) may be particularly applicable to the mouse Sirt1 gene, especially under starvation or HFD-fed conditions and in aging mice (Fig. 8).

Notably, although the two p53-responsive elements of the mouse *Sirt1* have been characterized to be functional in regulating *Sirt1* expression, the two mouse p53-responsive elements (−178 and −168 bp responsive elements both identified through the Genomatrix software package) did not show high sequence similarity to the well-established p53 binding consensus sequence (5′-WWWCGTGDDRRR-3′) of p53 target genes regulating cell cycle arrest and apoptosis in the previous studies^5,28^.

In this study, considering the initial observations that (1) abdominal adipose tissues were greatly reduced in old *Zbtb7c*-KO mice, (2) deletion of the Zbtb7c gene led to significant effects on lipid metabolism, and (3) the negative correlation between *SIRT1* and Zbtb7c expression, we investigated and proposed a model of how SIRT1 gene transcription might be regulated in mice fed an HFD and in aging mice. Intriguingly, Zbtb7c induced by an HFD and aging interacted with p53 and represses *SIRT1* expression, in which p53RE2 (which is closer to the ATG translation start codon) was more critical. Zbtb7c increased the binding of the corepressor mSin3A–HDAC complex to the p53–Zbtb7c complex at the p53REs, which potently repressed *SIRT1* gene transcription (Fig. 8).

Interestingly, in old *Zbtb7c* KO mice, abdominal adipose tissue mass was significantly reduced. The *SIRT1* promoter is thought to contain transcription regulatory elements important in SIRT1 expression and thereby adipocyte differentiation and adipogenesis. Zbtb7c mRNA expression has been shown to be increased by consumption of an HFD and aging. Aged *Zbtb7c-*KO mice in our study showed a decrease in abdominal fat tissue. Furthermore, microarray analyses of *Zbtb7c*-KO mouse liver and white adipose tissues revealed significant changes in the expression of genes involved in lipid metabolisms, such as *aP2*, *Fans*, *Cd36*, *Fsp27*, *Cpt1*, *Mcad*, *Pdk4*, and *SIRT1*. Together, these findings suggested that Zbtb7c is an important regulator of lipid metabolism. Previous studies indicated that SIRT1 directly interacted with and regulated the activity of two major transcription factors and coregulators of lipid metabolism, Pparγ, and Pgc-1α, via lysine deacetylation^2,26^ Because Zbtb7c represses *SIRT1*, it may also affect the expression of target genes such as Pparγ and Pgc-1α, as we demonstrated in *Zbtb7c*-KO MEFs (Fig. 6). Because deacetylation inactivates Pparγ but activates Pgc-1α, in our study, the target genes of Pparγ were repressed, and the target genes of Pgc-1α were upregulated by derepressed SIRT1 expression in the absence of Zbtb7c.

Zbtb7c, a potent transcriptional repressor of *Sirt1* under HFD feeding and aging conditions, may also affect various physiological processes regulated by Sirt1, such as cell survival, longevity, growth arrest, DNA repair, glucose metabolism, adipogenesis, inflammatory responses, and fatty acid oxidation. The results of our study suggested broad roles and the importance of Zbtb7c as a regulator of physiology. We were able to demonstrate that Zbtb7c is a regulatory protein linking stress, such as that induced by an HFD or aging, with *SIRT1* expression. We were able to identify a highly specific molecular target proximal promoter element (p53RE2) that can be engineered to control the expression of SIRT1 in the liver, pancreas, skeletal muscle, adipose tissues, etc., which can be beneficial in glucose and lipid metabolism, metabolic syndrome, pancreatic beta-cell function and insulin secretion, type 2 diabetes, longevity, etc.

## Supplementary information

Supplementary information
